# Fighting Fire With Fire: Oncolytic Virotherapy for Thoracic Malignancies

**DOI:** 10.1245/s10434-020-09477-4

**Published:** 2021-02-11

**Authors:** Chigozirim N. Ekeke, Kira L. Russell, Kyla Joubert, David L. Bartlett, James D. Luketich, Adam C. Soloff, Zong Sheng Guo, Michael T. Lotze, Rajeev Dhupar

**Affiliations:** 1 Department of Cardiothoracic Surgery, University of Pittsburgh School of Medicine, Pittsburgh, PA USA; 2 Department of Surgery, University of Pittsburgh School of Medicine, Pittsburgh, PA USA; 3 Departments of Immunology and Bioengineering, University of Pittsburgh School of Medicine, Pittsburgh, PA USA; 4 Veterans Affairs Pittsburgh Healthcare System, Surgical Services Division, Pittsburgh, PA USA

## Abstract

Thoracic malignancies are associated with high mortality rates. Conventional therapy for many of the patients with thoracic malignancies is obviated by a high incidence of locoregional recurrence and distant metastasis. Fortunately, developments in immunotherapy provide effective strategies for both local and systemic treatments that have rapidly advanced during the last decade. One promising approach to cancer immunotherapy is to use oncolytic viruses, which have the advantages of relatively high tumor specificity, selective replication-mediated oncolysis, enhanced antigen presentation, and potential for delivery of immunogenic payloads such as cytokines, with subsequent elicitation of effective antitumor immunity. Several oncolytic viruses including adenovirus, coxsackievirus B3, herpes virus, measles virus, reovirus, and vaccinia virus have been developed and applied to thoracic cancers in preclinical murine studies and clinical trials. This review discusses the current state of oncolytic virotherapy in lung cancer, esophageal cancer, and metastatic malignant pleural effusions and considers its potential as an emergent therapeutic for these patients.

Oncolytic viruses have been studied more than 70 years. The investigation was initiated with the pioneering work of Alice E. Moore at Memorial Sloan Kettering Cancer Center.^1^

Oncolytic virotherapy (OV) has a limited role for advanced cancer patients.^2^,^3^ Globally, few OVs are in regular use as approved treatment.

In October 2015, the first United States Food and Drug Administration-approved OV agent, talimogene laherparepvec (T-VEC), led the way for clinically effective virotherapy treatment of patients with metastatic melanoma.^4^,^5^ This genetically modified herpes simplex virus, encoding a human granulocyte macrophage colony-stimulating factor (GM-CSF) gene, is a lytic virus that promotes the release and presentation of tumor antigens to enhance an antitumor immune response both at the site of injection and systemically.^6^

In general, OV promotes immunity against neoepitopes, using the OV-mediated tumor cell lysis as an effective in vivo vaccination. Such immune-stimulating properties were initially demonstrated in murine tumor models^7^ and subsequently in clinical studies.^8^ Phases 1 and 2 studies demonstrated a direct oncolytic effect in injected tumors while also eliciting an immune-mediated anti-tumor response at non-injected sites.^8^

A phase 3 clinical trial of T-VEC demonstrated partial and complete responses at both injected and noninjected sites in patients with stage 3 or 4 disease (16.3%) compared with GM-CSF alone (2.1%).^4^ Before this, in 2005, H101 (Oncorine) was approved for the treatment of patients with late-stage refractory nasopharyngeal cancer in China.^9^ Oncorine (H101) is a recombinant human serotype-5 adenovirus (Ad5) with the E1B gene deleted, which leads to p53 inactivation and subsequently, viral replication. This virus and the related virus, ONYX-015, can replicate effectively in cancer cells with a dysfunctional (absent or mutated) p53 tumor suppressor gene.^10^ Further studies have indicated that late viral RNA export, rather than p53 replacement, determines ONYX-015 tumor selectivity.^11^ Currently, H101 is approved only in China for patients with advanced nasopharyngeal cancer.^9^,^10^

Finally, Rigvir is a genetically non-modified echovirus approved for treatment of melanoma.^12^ In a retrospective study, 298 patients with advanced melanoma who received adjunct intravenous Rigvir had an almost sevenfold increase in progression-free survival compared with patients observed after local excision.^12^

Another retrospective study assessed time to progression and overall survival for 79 patients with early-stage melanoma after treatment with Rigvir (*n* = 52) or observation alone (*n* = 27). The treatment arm had a four- to sevenfold lower mortality rate compared with that for patients who underwent observation.^13^ This treatment was previously approved in Latvia (2004–2019).^14^ Beyond these agents, OVs are relegated to preclinical studies and clinical trials. However, several promising OVs being considered for thoracic malignancies raise hope for wider adoption.

Many OV agents are genetically modified to limit replication within cancer cells while prohibiting replication in normal cells. The signaling pathways that are targeted comprise those that respond to hypoxia and include RB/E2F/p16, p53, protein kinase R (PKR), estimated glomerular filtration rate (EGFR), Ras, Wnt, apoptosis pathways, or interferon (IFN) and other innate immune-signaling pathways.^15^

This review describes the current state of OV in lung cancer, esophageal cancer, and metastatic malignant pleural disease, highlighting the potential benefits of broadening research and clinical trials that use these agents. Several current and ongoing studies that have used OV in murine models, and clinical trials demonstrate that these are promising options for patients with advanced disease (Tables 1 and 2).^16^–^18^

**Table 1:** Preclinical oncolytic virotherapy studies in thoracic malignancies

Virus	Viral designs	Studies by cancer type	Sample descriptions
Herpes simplex virus	AP27i145 HSV-1 (32,33);NV1066^77^	Preclinical: NSCLC^56^, gastric, esophageal cancer 77	(1) The combination of radiotherapy and AP27i145 infection was significantly more potent in killing NSCLC than each therapy alone. (2) NV1066 was tested in subcutaneous and intraperitoneal xenograft models; effective viral replication and oncolysis were evident in vitro (MOI, 0.1; an estimated 3500- ± 630-fold increase in viral production) and in vivo (a 73–77% decrease in tumor burden in the viral treated group vs phosphate buffered saline (PBS))
Vaccinia virus	VV.mIFNβ^63^, vvDD-IL2-RG, vvDD(67)	Preclinical: NSCLC^44^	VV.mIFNβ was able to slow tumor growth significantly in both models. VV.mIFNβ slowed tumor growth by ~ 40% (*p* < 0.05) after both systemic and intratumoral administration
Measles virus	MV-GFP^81^	Preclinical: MPE^81^	Intrapleural administration of 1.5 × 10^6^ PFU total dose of MV significantly improved median survival by approximately 80% vs the control animal group
Adenovirus	H101;Ad-Gfp^72^; Ad.p53^51^	Preclinical: NSCLC^72^; esophageal cancer^71^,^76^	(1) Intratumorally injected H101 virus suppressed growth of XWLC-05 (lung adenocarcinoma) in mouse models. (2) H101 for esophageal cancer yielded a 27% increase in overall response rates vs fluorouracil plus cisplatin-based chemotherapy alone(3)

HSV, herpes simplex virus; NSCLC, non-small cell lung cancer; PBS, phosphate buffered saline; VV, vaccinia virus; IFN, interferon; DD, double deletion; IL, interleukin; RG, rigid linker; MPE, malignant pleural effusion; PFU, plaque-forming unit; MV, measles virus; H101, Oncorine

**Table 2:** Clinical oncolytic virotherapy studies in thoracic malignancies

Virus	Viral designs	Studies by cancer type	Sample descriptions
Reovirus	Reolysin: Dearing strain of reovirus serotype 3	Completed: stage 4 NSCLC^50^ and chemorefractory stage 3 NSCLC^86^	Response rate for paclitaxel, carboplatin, and systemic reovirus was much higher (31%) than the historical response rate for paclitaxel and carboplatin alone (20%) in metastatic or recurrent NSCLC. In 37 patients with recurrent or metastatic NSCLC treated with reovirus in combination with paclitaxel and carboplatin median, PFS is 4 mos, median OS is 13.1 mos, and the 1-year OS rate is 57%
Senecavirus	Seneca Valley Virus (NTX-010)	Completed: SCLC^54^	50 Patients with SCLC and no progression of disease after four or more cycles of platinum-based chemotherapy were selected and randomized to receive a single dose of NTX-010 at 1.0 × 10^11^vp/kg or only saline (placebo). Median PFS was 1.7 mos for both the NTX-010 group and the placebo group; first-generation OV as a single agent could not generate obvious clinical responses in patients with advanced SCLC
Adenovirus	H101;Ad-Gfp^72^; Ad.p53^51^, Ad. IL2; Ad. b-gal; telomelysin; ColoAd1 (enadenotucirev)^49^	Completed: stages 3–4^51^ and unresectable stages 1–2 NSCLC;^14^ esophageal adenoCa^72^	The combination of rAd-p53 and BAI vs BAI alone in NSCLC patient is well tolerated and delays disease progression. The overall response rates (CR + PR) were 47.3% and 38.4% for the combo group and the control group, respectively (*p* < 0.05). IV enadenotucirev (ColoAd1) was shown to be safe for patients with resectable NSCLC
Herpes simplex virus	ADV/HSV-tk^72^	Ongoing: stages 3–4 NSCLC	Phase 2 trial that will determine the response rate, safety, OS, and morbidity of intratumoral ADV/HSV-tk and oral valacyclovir in the setting of SBRT (30 gry), followed by IV pembrolizumab for patients with chemo/immunotherapy-naïve locally advanced and metastatic NSCLC and triple-negative breast cancer
Adenovirus	OBP-301^75^; Ad-MAGEA3^87^	Ongoing: esophageal cancer; NSCLC	(1) Phase 2 trial that seeks to examine the clinical impact of OBP-301 in the setting of pembrolizumab as a third-line treatment for advanced gastroesophageal malignancy (2) Phases 1 and 2 dose-escalation trial of I.M Ad-MAGEA3, I.T MG1-MAGEA3 and IV pembrolizumab for patients with advanced NSCLC after one cycle of chemotherapy and/or anti-PD-1/PD-L1
Vaccinia virus	GL-ONC1^80^	Ongoing: MPE	Phase 1 trial seeking to assess the safety of dose escalation of intrapleurally administered GL-ONC1
Measles virus	TMV-108^88^	Ongoing: stages 3–4 esophageal cancer	Phases 1 and 2 trial to determine safety profile of TMV-018 in the setting of 5-FC vs anti-PD-1 for patients with advanced gastrointestinal tumors

HSV, herpes simplex virus; NSCLC, non-small cell lung cancer; Ad, adenovirus; MG1, Maraba-expressing MAGE-A3; AdenoCa, adenocarcinoma; ColoAd1, enadenotucirev; SCC, squamous cell carcinoma; vv, vaccinia virus; DD, double deletion; tk, thymidine kinase; RG, rigid linker; MPE, malignant pleural effusion; MV, measles virus; 5-FC, 5-fluorocytosine; PFS, progression-free survival; OS, overall survival; CR, complete response; PR, partial response

We divided the review into the following subcategories: immunologic principles of OV and individual tumor presentations (lung cancer, esophageal cancer, and malignant pleural effusion). We purposely excluded mesothelioma because a few excellent recent reviews have analyzed immunotherapy and oncolytic virotherapy for this type of cancer.^19^,^20^

## Immunologic Principles Underlying Oncolytic Virotherapy

The development of oncolytic viruses aims to promote targeted immunogenic cell death while minimizing damage to normal tissues. Stimulating the host immune system and overcoming an immunosuppressive tumor microenvironment (TME) through viral infection, particularly with the ability to deliver cytokine payloads, holds great potential for future therapies, particularly in the setting of combined multi-modality therapeutics.^21^

Our group has assessed many individual chemokines and cytokines in murine models delivered by oncolytic vaccinia virus including interleukin (IL)-2 and IL-15, both of which appear promising for use in thoracic malignancies given the high mutational load and potential neoepitopes conferring immunogenicity. Given the systemic toxicity of these gamma-common chain cytokines, providing them as cell surface-bound molecules with viruses that preferentially are expressed in tumors has great promise.

Extrinsic agents that appear to have potential use are small molecule inhibitors of p38, CBL-B, and AKT as well as the antibody checkpoints described in this review.^22^,^23^

Viral modification allows for selective infiltration and proliferation in tumor cells while largely sparing healthy host cells. These modifications often include deletions to the viral genome of genes preferentially expressed in malignant cells, a form of so-called synthetic lethality.^15^,^24^ Highly immunogenic OV can promote local immunity through resultant interferon-mediated upregulation of major histocompatibility complex I (MHC I) molecules as well as adhesion molecules to improve antigen presentation.^25^ Dendritic cell (DC) recruitment across activated endothelium, loading of antigen, and maturation occurs in response to release of damage-associated molecular pattern molecules (DAMPs), including high-mobility group box 1 (HMGB1), extracellular adenosine triphosphate (ATP), and calreticulin exposure on the tumor cell surface.^26^ The recruitment and maturation of DCs lead to the priming of T cells in secondary or tertiary lymphoid sites.^27^ Pro-inflammatory cytokines released within the TME also can upregulate prostaglandin production and immunosuppressive molecules associated with an increased presence of myeloid-derived suppressor cells.^28^

**Fig. 1:**
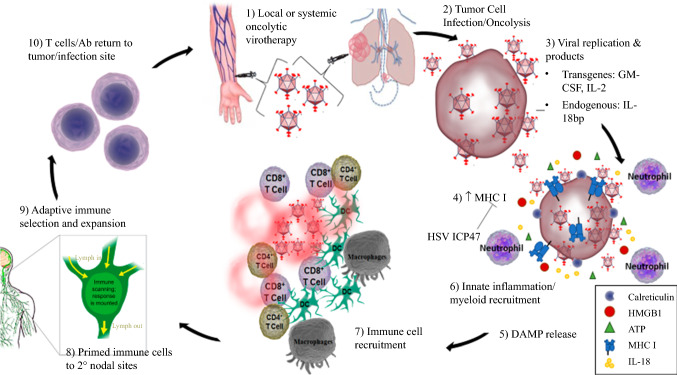
Oncolytic virotherapy-mechanism of action. An oncolytic virus is administered locally or systemically (1), thus targeting tumor cells while sparing normal tissue (2). Viral replication (3) promotes MHC I expression (4) and DAMP release (5) with a subsequent innate immune response (6,7). Immune cells are primed in the lymphatic system (8), leading to an adaptive immune response (9) and further tumor lysis (10). MHC I, major histocompatibility complex I; DAMP, damage-associated molecular pattern molecule; HMGB1, high molecular group box-1

Infection activates antigen-presenting cells, recruits critical adaptive immune cells, and most notably, promotes adaptive immune cell survival through nuclear translocation of nuclear factor kappa-light-chain-enhancer of activated B cells (NF-κB).^29^ This in turn leads to the production of various chemokines and cytokines responsible for neutrophil recruitment and enhanced T cell responses. Several viral gene products that inhibit this pathway, such as those encoded within the vaccinia virus (VV), must be modified to elicit an effective pro-inflammatory response.^29^ The A52, B15, and K7 gene products represent a few of the poxvirus NF-κB pathway products that together inhibit this pathway. Deletion of these genes or others targeting various mechanisms within the NF-κB pathway can influence NF-κB activation and downstream neutrophil recruitment.^30^ The vector must stimulate an effective inflammatory response that will clear and/or control the infected tumor through the elicitation of adaptive immunity but also allow for viral replication to target tumor cells (Fig. 1).^30^

Immunodominant epitopes pose an inherent obstacle to promotion of an immunogenic response after oncolytic virotherapy administration. Tumor-associated antigens (TAAs) must compete with viral proteins that are intrinsically more immunogenic.^31^ This imbalance yields disproportionate proliferation and accumulation of adaptive immune cells targeting the viral proteins and antigens, with only limited targeting of the weaker TAA epitopes. To overcome this obstacle, one method under investigation uses viral innate MHC inhibitors. Other studies have prioritized strengthening TAA immunogenicity by using viruses that express multiple human antigens and costimulatory molecules or by pseudotyping the virus via attachment of tumor antigen onto the expressed viral envelope.^32^,^33^

Viruses also can beneficially mediate epitope-spreading to enhance recognition of tumor antigens. Attraction to infected target cells increases recruitment of T cells that recognize tumor antigens, resulting in epitope-spreading and recognition of a broader range of targets, including the TAAs that dissociate from the expressed viral proteins.^34^

Another factor that has an impact on viral infection and host response is the lymphatic system. The lymphatic vessels form an active barrier that can decrease fluid transport and minimize viral dissemination, acting as an “innate-like” component of the host defense system. In metastatic malignant pleural disease, dysfunction of the lymphatic system may serve to limit the spread of oncolytic virus, resulting in retained fluid within the pleural space.^35^ This also poses the potential disadvantage of limiting transport of antigen to tertiary lymphoid structures for immune induction. However, research on viral dissemination and immune activation has shown that lymphatic vessels differentially regulate fluid and cell transport secondary to local interferon production, leading to viral sequestration while promoting dendritic cell maturation and migration to nodal sites.^36^ Lymphatic dysfunction decreases lymph-dependent immune induction and contributes to persisting pathology. Thus, lymphatics play a critical role in regulating viral infection, both by minimizing its spread throughout the body and by promoting an adaptive immune response,^37^,^38^ allowing us to consider that they play a major role in limiting fluid egress from the thoracic cavity in the setting of benign and malignant pleural effusions.^39^

The following sections have been divided by the major epithelial thoracic malignancies for which OVs might be used (lung cancer, esophageal cancer, and metastatic malignant pleural effusions) in both preclinical studies and clinical trials. In addition to single-agent therapy, combination therapies (e.g., checkpoint inhibitors with OV) are included because they have shown promise, with potential to modulate the immunologic TME and promote a more effective host response.^40^,^41^ Additionally, the widespread use of checkpoint inhibitors has energized an immunotherapy renaissance in solid tumor treatment strategies and likely will continue as a mainstay of treatment. Cytotoxic T lymphocyte-associated antigen 4 (CTLA-4), programmed death-1 receptor (PD-1), and programmed death ligand 1 (PD-L1) blocking monoclonal antibodies are in widespread use for some of these patients.^42^–^44^

## Lung Cancer: Clinical Trials of Oncolytic Virotherapy

Non-small cell lung cancer (NSCLC) and small cell lung cancer (SCLC) are leading causes of cancer-related mortality among both men and women, and they remain among the most common cancers worldwide.^45^ Globally, lung cancer accounts for 13% (1.6 million) of the total cancer cases and 18% to 25% of cancer-related deaths.^45^

Lysogenic adenovirus has an extensive tissue tropism and the capability of infecting a large variety of dividing and non-dividing cells.^46^ A two-armed clinical trial involving 58 patients was conducted to evaluate an oncolytic adenovirus (rAd-p53) as adjunct therapy to bronchial arterial infusion chemotherapy in the treatment of patients with stage 3 or 4 NSCLC.^47^ In this trial, 33% of the patients received a combination of Ad-p53 injection (via intra-tumoral or bronchial artery access) and bronchial artery instillation of chemotherapy (fluorouracil, navelbine, and cisplatin), with the remaining patients receiving only the bronchial artery instillation of chemotherapy. The patients in the combination group had a longer time to progression than those in the control group (median, 7.75 vs 5.5 months; *p *= 0.018). Interestingly, a complete response was observed in two patients with stage 3 NSCLC in the Ad-p53 arm of the study. Overall, Ad-p53 gene transfer is well tolerated by patients, with minimal side effects but no change in survival.

A phase 1 clinical trial demonstrated the feasibility and safety of intravenous enadenotucirev for 12 patients with resectable NSCLC. Enadenotucirev is a chimeric adenovirus that mediates tumor-selective cytotoxicity through direct non-apoptotic, pro-inflammatory cell-killing mechanisms.^43^ The treatment period included a single cycle of OV (3 × 10^11^ viral particles [vp]) followed by surgical excision of residual disease. Enadenotucirev has a low immunogenic profile but has demonstrated a pro-inflammatory response (increased CD8 + T cell activation) after administration.^48^ The pre- and post-dose cytokine responses were equivalent and not associated with adverse events related to the OV. This is one of few studies that highlight the safety profile of intravenously administered oncolytic viruses.^49^

Reolysin is a wild-type, unmodified Dearing-strain reovirus, a stable, nonenveloped double-stranded RNA virus. It has anticancer activity against multiple malignancies (breast, colon, bladder, pancreas, lung, and esophageal cancers).^50^ In a phase 2 study, the Response Evaluation Criteria in Solid Tumors (RECIST) response rate for paclitaxel and carboplatin in combination with systemic Reolysin was much higher (31%) than the historical response rate of paclitaxel and carboplatin alone (20%) for patients with metastatic or recurrent NSCLC.^51^ Four patients with stable disease had more than a 40% reduction in positron emission tomography (PET) standardized uptake values. Seven patients were alive after a median follow-up period of 34.2 months, and two of these patients were without disease progression at respectively 37 and 50 months.

Seneca Valley Virus isolate 001 (SVV-001, now NTX-010) is a virus from the genus Senecavirus, family *Picornaviridae*. In 2007, two novel discoveries on the potential of SVV-01 as an OV were made.^52^ First, neuroendocrine tumor cells are much more sensitive to the cytotoxicity of this picornavirus than any adult normal human cells tested. Second, the viral infectivity was not inhibited by human blood components, suggesting that this OV could be delivered intravenously. These findings in addition to those of follow-up basic studies set up a solid foundation for clinical trials with this nonpathogenic OV.

Rudin et al.^53^ evaluated Senecavirus in patients with neuroendocrine-type cancers in a phase 1 study. These authors performed single intravenous doses up to 1.0 × 10^11^ viral particles (vp)/kg and showed that even the highest dose of the virus was well tolerated, with predictable virus clearance kinetics and intra-tumoral replication in SCLC and other cancers. In their randomized, double-blind, placebo-controlled phase 2 clinical study recently published in 2020,^54^ 50 patients with extensive-stage SCLC without progression of disease after four or more cycles of platinum-based chemotherapy were selected and randomized to receive a single dose of NTX-010 at 1.0 × 10^11^vp/kg or saline (placebo). Progression-free survival (PFS) was set as the primary end point, followed by viral clearance and detection of neutralizing antibodies. The specified interim analysis unfortunately indicated that the median PFS was 1.7 months for both the NTX-010 group and the placebo group. Thus, the trial was terminated due to its futility. This study showed that a first-generation OV as a single agent was unable to generate obvious clinical responses in these patients.

## Lung Cancer: Preclinical Studies of Oncolytic Virotherapy

Oncolytic herpes simplex virus, a double-stranded DNA virus (~ 152 kb), has been studied in several in vitro tumor models. The incorporation of microRNA-mediated regulation of key viral genes, such as miRNA-145, has promoted in vitro cytotoxicity in several NSCLC cell lines. The tumor suppressor, miRNA-145, is downregulated in lung cancer and several other solid tumors (e.g., colon, ovarian, and prostate tumors). Higher expression of miRNA-145 is found in normal human cell lines (human umbilical vein endothelial cells [HUVEC], pneumonia/heart failure associated pleural effusions [PL2, PL1]) than in human NSCLC cell lines (A549, H460, H838, and H197). The cytotoxic impact of miRNA145-regulated ICP27 oncolytic HSV-1 expression was tested in vitro.

The multifunctional regulatory protein, ICP27 (regulated by miRNA145), is required for herpes simplex virus type 1 (HSV-1) infection. Four copies of the miRNA145 target sequence were incorporated into the 3′-untranslated region of ICP27 to create an AP27i145 amplicon virus. The virulent impact was compared with that of a replication-deficient recombinant ICP27^−^ helper virus, *5dl1.2,* because it lacks the ICP27 gene and cannot replicate alone.^55^

In A549, H460, H838, and H1975 NSCLC cell lines, the survival of the AP27i145-infected cells was significantly lower than that of the *5dl1.2*-infected cells at an MOI of 0.1 on post-infection day 5 (*p* < 0.05 in all comparisons). The cytotoxicity of AP27i145 was significantly stronger than that of *5dl1.2,* at an MOI of 0.01 in A549 and H460 cells (*p* < 0.05 in both comparisons). However, no significant difference in survival was found when AP27i145 and *5dl1.2* were applied to normal human cell lines. These findings suggest that AP27i145 may have selective cytotoxicity in NSCLC cell lines and may be of potential value.^55^,^56^

Many studies have used oncolytic adenovirus (oAdv) variants in lung cancer models, and several strategies have been used to improve the efficacy of these viruses. For example, an oAdv with a promoter-dependent telomerase that expresses HSV-TK showed potency and safety both in vitro and in vivo.^57^ The ability of oAdv to eliminate cancer stem cells often refractory to conventional chemo/radiotherapies may provide inhibition of cancer recurrence and metastasis.^58^–^60^

Coxsackievirus B3 (CVB3) possesses specific oncolytic activity against nine human NSCLC cell lines. In vitro, CVB3 induced cell apoptosis and cell survival, signaling pathways associated with phosphoinositide 3-kinase/Akt and mitogen-activated protein (MAP)/extracellular signal-regulated (ERK) kinase (MEK) pathways, leading to cytotoxicity and regulation of CVB3 replication. In transplantable lung tumor models, intralesional injections of the virus led to remarkable regression. Virus-infected NSCLC cells also expressed abundant cell surface calreticulin, secreted ATP, and translocated extranuclear HMGB-1, which are DAMP molecules that mark immunogenic cell death (ICD).^6^,^61^

Oncolytic vaccinia viruses (OVVs) with three individual genetic backbones have been applied preclinically. Some investigators have used thymidine kinase (TK) deletion alone, whereas others have used viruses with dual deletion of viral genes encoding both TK and the viral growth factor B18R– or dual deletion of viral genes encoding TK**–** and viral growth factor (VGF).^62^ These genetic modifications aim to enhance tumor selectivity while retaining potency when infecting tumor but not normal cells.^15^,^30^

An OVV was used as a vector to deliver interferon beta (IFNβ) directly into tumors. An oncolytic VV mutant (TK-/B18R-/INFβ +) (VV.mIFNβ) was used in subcutaneous murine models of the NSCLC cell lines, TC-1 and LKRM2. In both models, VV.mIFNβ slowed tumor growth significantly by approximately 40% (*p *< 0.05) after either systemic or intra-tumoral administration. Interestingly, the mechanism of tumor destruction was distinctly different for each route of administration. In the LKRM2 line, the mechanism of in vivo cytotoxicity was secondary to induction of a local inflammatory response, with infiltration of CD8^+^ T cells after intra-tumoral and systemic administration. In contrast, a direct oncolytic effect was primarily responsible in the TC-1 line.^63^

An OVV with the tk gene deleted but expressing IL-24 (VV-IL-24) efficiently infected A549 human lung cancer xenografts, promoting caspase-dependent apoptosis with decreased STAT3 expression.^64^ Interleukin-24 (IL-24)/mda-7 is a member of the IL-10 family of cytokines that signals through two receptors (IL-20R1/IL-20R2 and IL-22R1/IL-20R2). Unlike other IL-10 family members, it inhibits STAT3 expression, thus promoting apoptosis. This virus had demonstrable efficacy in a syngeneic murine model using transplantation of Lewis lung cancer cells.^64^

Use of bi- and tri-specific T cell engager-armed OVs may indeed be the next advance in cancer immunotherapy.^65^ An OVV was created that encoded a secretory bispecific T cell engager consisting of two single-chain variable fragments for CD3 as well as the tumor cell surface antigen EphA2 (EphA2-TEA-VV).^66^ Tumor cells infected with EphA2-TEA-VV induced T cell activation. In vitro, EphA2-TEA-VV not only lysed infected tumor cells, but also induced bystander killing of noninfected tumor cells when in the presence of T cells.^66^ In a lung cancer xenograft model, EphA2-TEA-VV-infected T cells had potent antitumor activity compared with control VV-infected T cells. These findings provided a new strategy using T cell engager-armed oncolytic virus for enhanced cancer immunotherapy.^66^

Recently, our group created an OVV used to deliver membrane-bound phosphoinositol glycan-linked IL-2 into the TME.^67^ This allowed local delivery of IL-2 into tumor tissues with reduced systemic toxicity. The techniques tested for membrane association of IL-2 included use of a transmembrane glycosylphosphatidylinostitol anchor with either a rigid linker (RG) or a flexible linker (FG). Intraperitoneal injection with vvDD-IL-2 but no linker led to IL-2 toxicity, with serum IL-2 levels 100 times higher than mice treated with membrane-bound forms. However, vvDD-IL-2-FG and vvDD-IL-2-RG significantly extended survival, with vvDD-IL-2-RG having the greatest impact on survival, substantially decreasing tumor volume in murine colon, lung, and ovarian cancer models. The antitumor effect elicited by vvDD-IL-2-RG treatment was found to be IFN-γ and CD8^+^ T cell dependent, and surprisingly NK depletion enhanced antitumor effects. Subcutaneous murine Lewis lung carcinoma tumors were successfully treated with complete eradication by day 15, whereas persistent disease was evident in the PBS cohort.^67^ We have found that intrapleural injection has greater anti-tumor efficacy than systemic treatment with enhanced diversity in the recruited T cell repertoire.^68^

## Esophageal Cancer: Clinical Trials of Oncolytic Virotherapy

For patients with early-stage esophageal cancer, improved survival has been credited to earlier detection, improved surveillance, and minimally invasive methods of surgical resection. However, advanced esophageal cancer continues to be a difficult problem, with low a 5-year survival rate (~ 20%) despite improved locoregional and systemic treatments.^69^–^71^ Oncolytic virotherapy has been studied as part of the multi-modal treatment for patients with esophageal cancer.

A multicenter randomized phase 3 clinical trial showed that the addition of the adenovirus H101 to fluorouracil (5-FU) plus cisplatin-based regimens for head and neck squamous cell carcinoma and esophageal cancer yielded a 27% increase in overall response rates.^72^

Recently, a preclinical study using a murine orthotopic esophageal cancer xenograft model demonstrated that intra-tumoral injection of the adenovirus telomelysin plus regional irradiation induced tumor cell-specific radiosensitization, which has prompted phases 1 and 2 clinical trials.^73^ An ongoing phase 1 study will assess the safety and tolerability of intra-tumoral telomelysin in combination with radiation therapy for patients with unresectable esophageal cancer,^74^ whereas a separate phase 2 study will assess intratumoral telomelysin and systemic pembrolizumab for patients with advanced esophageal adenocarcinoma who have failed two prior lines of therapy.^75^

## Esophageal Cancer: Preclinical Studies of Oncolytic Virotherapy

A third-generation, replication-competent oncolytic herpesvirus containing transgenes encoding GALV and *Fcy:Fur* was applied in murine gastroesophageal cancer models.^76^ The herpes viral construct includes a double deletion of the *γ*_*13*_*4.5* gene as well as a single deletion of the *US12* gene, allowing for tumor specificity while enhancing an immune response. Viral cytotoxicity and replication were tested in gastroesophageal cell lines OCUM-2MD3 (gastric adenocarcinoma), MKN-45 (gastric adenocarcinoma), AGS (gastric adenocarcinoma), MKN-1 (gastric adenosquamous carcinoma), MKN-74 (gastric adenocarcinoma), and BE-3 (esophageal adenocarcinoma) at serial multiplicities of infection. The BE-3 cell line showed 95% cytotoxicity by day 5 and 74% cytotoxicity by day 7, with an MOI of 0.1.^76^ In Barret’s esophagus cell lines, NV1066, a replication-competent attenuated HSV-1 mutant virus, has been tested in vivo (in intraperitoneal and subcutaneous mouse models) and in vitro. Intra-tumoral injection of NV1066 decreased progression of subcutaneous tumors by 77% at 4 weeks compared with PBS-treated mice (*p *< 0.001). Intraperitoneal injection of NV1066 decreased tumor burden by 73% after 4 weeks versus treatment with PBS alone (*p *< 0.001).^77^

## Metastatic Malignant Pleural Effusions: Clinical Trials of Oncolytic Virotherapy

Metastatic malignant pleural effusions (MPEs) are diagnosed for more than 200,000 patients annually in the United States, most commonly for patients harboring NSCLC (36.0%) or breast carcinoma (26%).^78^ The mainstay of MPE treatment is drainage with or without pleurodesis.^43^ Very rarely, debulking surgery with pleurectomy and intrathoracic chemotherapy is used. Treatment is primarily palliative, and the mortality rate has remained largely unchanged during the past 20 years.^78^,^79^

Intrapleural talimogene laherparepvec will be tested in a phase 1 clinical trial for MPE secondary to NSCLC in combination with systemic nivolumab.^30^

A phase 1 study investigating intrapleural administration of the OVV GL-ONC1 has been completed for patients with malignant pleural effusions. The study analyzed 11 patients with MPE, and intrapleural administration of GL-ONC1 was deemed safe but best suited for patients with MPM whose disease is limited to the pleura space.^80^ Currently, no clinical studies have demonstrated efficacy of intrapleural OV for non-mesothelioma MPE.

## Metastatic Malignant Pleural Effusions: Preclinical Studies of Oncolytic Virotherapy

Measles virus (MV) was applied in an MDA-MB-231 (breast cancer) murine pleural effusion model.^81^ Viral replication and syncytia formation were assessed after systemic and intrapleural administration. Two days after inoculation at an MOI of 1.0, MV-GFP resulted in 100% infection of MDA-MB-231 monolayers with formation of giant multinucleated syncytia. Cell viability was reduced by approximately 50% at 48 h. Intrapleural administration of 1.5 × 10^6^ plaque-forming units significantly improved median survival compared with the control group (54.5 vs 30.5 days; *p* = 0.001).

A subcutaneous PC14PE6 lung adenocarcinoma orthotopic xenograft in athymic mice develops subcutaneous malignant effusions (MEs) which mimic MPEs. Localized OVV encoding of a single-chain antibody against vascular endothelial growth factor (VEGF) had a significant therapeutic effect for both advanced lung adenocarcinoma and the subcutaneous ME.^82^

## Conclusions and Perspectives

Oncolytic virotherapy has reached the stage of clinical trials, with a few OVs already approved for treatment. One major lesson learned from clinical trials is that OV monotherapy has limited efficacy. Oncorine (H101) in combination with chemotherapy is approved for patients with nasopharyngeal carcinoma in China, and Imlygic(T-VEC) is approved to treat stage 3b IVM1c melanoma in the United States, Europe, and Australia. In both cases, the efficacy has been rather limited. Therefore, like other types of cancer therapeutic agents, OVs may need to be applied in combination to enhance therapeutic efficacy while maintaining tolerable toxicities. The consensus in the field has been that OVs are an excellent platform for combination therapy to treat cancer patients.^3^,^83^–^85^ A rational combination could be OVs administered with immune checkpoint blockade.

Another practical consideration, especially for thoracic cancers, is how to optimize delivery when a localized strategy is preferred. Some sites, such as the pleural cavity, might be relatively isolated from intravenously administered therapies. Local delivery has not been popular due to the need for an invasive procedure. However, because the pleural cavity can readily and repeatedly be accessed with indwelling catheters (often placed as standard of care), straightforward options exist but may not often be considered. Lung parenchymal nodules present a separate challenge, and accessing these for directed injections requires either radiologic or bronchoscopic procedures, both of which may be inaccurate. Fortunately, advances in technology, such as navigational robotic bronchoscopy, have opened opportunities for safe and accurate delivery of treatments (immunotherapies or ablative therapies) to nodules in the lung parenchyma that have previously been deemed too difficult to reach.

The ability of OVs to target disseminated tumor cells and treat refractory disease while also having a low side-effect profile may make them a favorable immunotherapy method. Regarding its application in thoracic cancers, oncolytic viro-immunotherapy has to date been studied in preclinical models and early-stage clinical trials only. We predict that combination regimens may be more successful for patients with advanced thoracic malignancies, but additional preclinical and clinical studies need to be performed to demonstrate the role of OV in that capacity.
